# mTORC1 Mediates the Processes of Lysine Regulating Satellite Cells Proliferation, Apoptosis, and Autophagy

**DOI:** 10.3390/metabo12090788

**Published:** 2022-08-25

**Authors:** Mengqi Liu, Zhengkai Yue, Bin Zhang, Fan Li, Lei Liu, Fuchang Li

**Affiliations:** 1 Department of Animal Science and Technology, Shandong Agricultural University, Tai’an 271018, China; 2 Department of Animal Science and Technology, Henan Agricultural University, Zhengzhou 450002, China; 3 Henan Key Laboratory of Innovation and Utilization of Grassland Resources, Zhengzhou 450002, China; 4 Henan Forage Engineering Technology Research Center, Zhengzhou 450002, China

**Keywords:** lysine, satellite cells, proliferation, apoptosis, autophagy, mTORC1 signaling pathway

## Abstract

Lysine (Lys) is essential for skeletal muscle growth and protein synthesis in mammals. However, the regulatory network underlying Lys-regulated skeletal muscle development is unknown. To determine whether any cross-talk occurs among mammalian targets of rapamycin complex 1 (mTORC1) and Lys in the regulation of muscle satellite cells (SCs) proliferation, we applied the treatment rapamycin (a mTORC1 inhibitor) and MHY1485 (a mTORC1 activator) on Lys-added or -deficient SCs. The results show Lys deprivation significantly decreases SCs viability, protein synthesis, and cell cycling, increases autophagy and apoptosis, and inhibits the mTORC1 signaling pathway. Restoration of Lys content significantly attenuates this effect. mTORC1 signaling pathway activation during Lys deprivation or mTORC1 signaling pathway inhibition during Lys addition attenuates the effect of Lys deprivation or addition on SCs viability, protein synthesis, cell cycling, autophagy, and apoptosis. In conclusion, Lys could improve SCs proliferation, and inhibit SCs apoptosis and autophagy, via the mTORC1 signaling pathway.

## 1. Introduction

Muscle growth is driven by muscle stem cells, which can eventually form the most basic structures of skeletal muscle. Muscle SCs are mainly found in the basement membrane and myofibrils of muscle fibers [1]. Skeletal muscle is mostly composed of multinucleated myofibers after mitosis, and myogenesis is a multistep process that includes activation, proliferation, differentiation, and formation of multinucleated myofibers in muscle SCs [2]. The mTORC1 signaling pathway plays an important role in skeletal muscle regeneration [3]. mTORC1 consists of mTOR, raptor, mlST8, and two negative regulatory structures, PRAS40 and Deptor, which regulate cell growth by promoting translation, ribosomal biosynthesis, and autophagy [4]. The phosphorylation of the mTORC1 signaling pathway is necessary for the proliferation, differentiation, and activation of SCs [5,6]. In addition, insulin-like growth factor-1 promotes SCs proliferation and reduces autophagy by increasing the phosphorylation level of mTORC1 and its downstream proteins [7]. Moreover, overexpression of muscle blind-like 1 protein inhibits autophagy by regulating phosphorylation levels of the mTORC1 signaling pathway, reversing the defective proliferation of skeletal muscle SCs in myotonic dystrophy [8].

Lys is the first limiting amino acid (AA) for most mammals, which could promote muscle protein synthesis and hypertrophy of muscle fibers [9,10]. Lys could inhibit some muscle fiber protein degradation through the autophagosome–lysosome system, and maintain body protein stability [11]. Severe Lys restriction in mice adversely affects both body growth and the regulation of lipid and nitrogen metabolism [12]. A study shows that Lys supplementation inhibits autophagic activity and reduces muscle protein loss in aging mice [13]. Lys deficiency can also contribute to apoptosis [14]. In our previous study, supplementation with moderate amounts of Lys increases daily weight gain and improves skeletal muscle development [15]. Similar to our study, a study shows that Lys deficiency leads to decreased protein synthesis and diminished cell proliferation in SCs [16]. However, the potential link between the effect of Lys on muscle growth and the mTORC1 signaling pathway is unclear.

mTORC1 is a conserved serine/threonine (Ser/Thr) kinase, which is recognized as the main AA receptor [17]. In recent years, many studies have shown that Lys is an important signaling molecule and it affects the mTORC1 signaling pathway [18]. Many functions of the mTORC1 signaling pathway are inferred by the inhibition of the mTORC1-specific inhibitor rapamycin. As the first clinical mTORC1 inhibitor rapamycin is a natural antibiotic, it does not directly inhibit mTORC1 activity, but through the formation of complexes with mTORC1 domains plays the role of an allosteric inhibitor [19]. MHY1485 is a new type of specific mTORC1 activator of the cellular permeable small molecule. The compound directly binds to mTORC1, activates mTORC1 at μM concentrations, and significantly increases its activity [20].

In the current work, we compared our research with previous studies [13,14,15,16]. We investigated the effect of Lys on SCs proliferation, protein synthesis, and the mTORC1 signaling pathway by precisely controlling the level of Lys in the cell culture medium, and demonstrate the function of the mTORC1 signaling pathway in Lys regulating SCs metabolism. In addition, we explored the link between autophagy and apoptosis with the mTORC1 signaling pathway and Lys.

## 2. Materials and Methods

### 2.1. Isolation and Identification of SCs

Single muscles were first isolated from the dorsolumbar muscles of 3 day old rabbits (3 rabbits), excess fat and connective tissue were cut off with ophthalmic scissors, and the muscles were cut into minced meat and digested in 0.3 mg/mL collagenase I (Sigma, Aldrich, St. Louis, MO, USA) and 0.25% trypsin–ethylene diamine tetraacetic acid (Trypsin–EDTA) (Gibco, Carlsbad, CA, USA) for 60 min before the addition of Dulbecco’s modified eagle medium/F-12 (DMEM/F12, Gibco, Carlsbad, CA, USA) containing 10% fetal bovine serum (FBS, Invitrogen Corporation, Carlsbad, CA, USA) to terminate the digestion. Tissue debris with cell suspension was collected by passing the cells through a 70 μm cell sieve, centrifuged at 1200 rev/min for 8 min at room temperature. The supernatant was then removed, and the cells were initially resuspended by adding complete medium to obtain a cell suspension. Cells were suspended in selection medium containing 15% FBS, 10 ng/mL basic fibroblast growth factor (BFGF), 10 ng/mL epidermal growth factor (EGF), 2 mM/L L-glutamine, and 1% penicillin–streptomycin in DMEM/F12 (all from Invitrogen Corporation, Carlsbad, CA, USA). The cell suspensions were inoculated into T25 culture flasks and cultured at 37 °C in 5% CO_2_ for 2 h. Then, later, the suspensions were transferred onto new plates, and differential apposition was repeated 3–4 times to remove a large number of fibroblasts, labeled as P0 generation cells, and SCs started to culture for about 24 h. The culture was maintained until confluency reached about 80%, and the cells were then washed twice with sterile phosphate buffer solution (PBS, Gibco, Carlsbad, CA, USA), dissociated by 0.25% trypsin–EDTA. The cells were passaged onto new plates at a ratio of 1:2. As the number of passages increased, the cells continued to proliferate.

P0 generation cells were inoculated into six-well microplates for immunofluorescence and induction of differentiation identification. Anti-desmin, anti-myogenic differentiation (MyoD), anti-paired box 7 (Pax7), and anti-myosin heavy chain (MYHC) were used; all antibodies came from Cell Signaling Technology (Danvers, MA, USA). Immunofluorescent detection was performed as previously described [21].

### 2.2. Effect of Lys on SCs Proliferation, Apoptosis, and Autophagy (Experiment 1)

**Figure 1:**
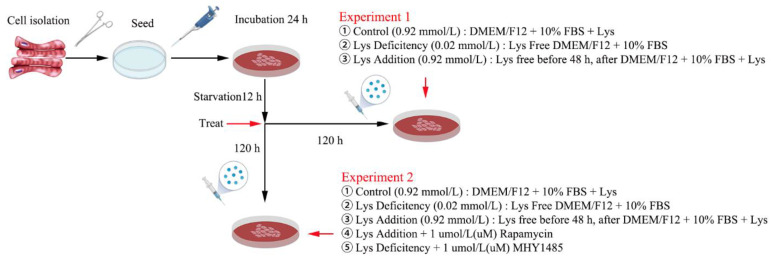
Test procedure.

Cells adhered to the wall after inoculation for 24 h, then starved for 12 h in the medium without Lys and FBS, and divided into three groups: cultured in the medium with 0.92 mmol Lys for 120 h (Control, CON); cultured in the medium with 0.02 mmol Lys for 120 h (Lys deficiency, LD); cultured in the medium with 0.02 mmol Lys for 48 h, and then cultured in the medium with 0.92 mmol Lys for 72 h (Lys addition, LA). The cell proliferation was measured at 24, 48, 72, 96, and 120 h, and the cell samples were collected at 120 h (Figure 1).

### 2.3. Function of the mTORC1 Signaling Pathway on Lys Regulating SCs Proliferation, Apoptosis, and Autophagy (Experiment 2)

Cells adhered to the wall after inoculation for 24 h, and were then starved for 12 h in the medium without Lys and FBS, and divided into five groups: cultured in the medium with 0.92 mmol Lys for 120 h (Control, CON); cultured in the medium with 0.02 mmol Lys for 120 h (Lys deficiency, LD); cultured in the medium with 0.02 mmol Lys for 48 h; and then cultured in the medium with 0.92 mmol Lys for 72 h (Lys addition, LA); cultured in the medium with 0.02 mmol Lys for 48 h, and then cultured in the medium with 0.92 mmol Lys and 1 μM rapamycin for 72 h (Lys addition + 1 μM Rapamycin, LAR); cultured in the medium with 0.02 mmol Lys for 48 h, and then cultured in the medium with 0.02 mmol Lys and 1 μM MHY1485 for 72 h (Lys deficiency + 1 μM MHY1485, LDM). Cell proliferation was measured at 120 h and the cell samples were also collected at 120 h (Figure 1).

### 2.4. Cell Proliferation Assay

For the Cell Counting Kit-8 (CCK8, Sigma, Aldrich, St. Louis, MO, USA) method, 20 μL of water-soluble tetrazolium salt was added to each well, incubated for 1 h at 37 °C in a constant temperature incubator, and then removed and restored to room temperature. The OD of each well was measured at 450 nm using a microplate reader (Bio-Rad, Hercules, CA, USA).

### 2.5. Flow Cytometry

Cell cycle progression and apoptosis were measured using PI/RNase standing buffer (BD Biosciences, cat: 550825, New York, NY, USA) and PE Annexin V Apoptosis Detection Kit I (BD Biosciences, cat: 559763, New York, NY, USA), respectively, with flow cytometry (BD Accuri C6, BD, New York, NY, USA). The results were analyzed with ModFit LT 5.0 software (5.0.0.0, Verity Software House, San Francisco, CA, USA).

### 2.6. Western Blotting

Total protein was extracted from SCs using a radioimmunoprecipitation assay (RIPA) lysis buffer (Beyotime, Shanghai, China), and the protein concentrations were determined using a bicinchoninic acid assay (BCA) protein assay Kit (Biotechnology, Beijing, China). The extracted proteins (50 ng/sample) were solubilized in 40 mL of sodium dodecyl sulfate (SDS) loading buffer (Solarbio, Shanghai, China) and then resolved by electrophoresis (Bio-Rad, Hercules, CA, USA) on 12.5% SDS-polyacrylamide gel electrophoresis (PAGE) gels prior to electrophoretic transfer to polyvinylidene fluoride (PVDF) membranes (Millipore, Billerica, MA, USA). Standard markers for protein molecular masses were purchased from Thermo (Waltham, MA, USA). The proteins were closed in fast closing solution at 37 °C for 30 min and incubated with primary antibodies at 1:2000 dilution (anti-mTOR; anti-phospho-mTOR, Ser2448; anti-ribosomal protein S6 kinase I (P70S6K1); anti-phospho-P70S6K1, Thr389; anti-ribosomal protein S6 (S6); anti-phospho-S6, Ser235/236; anti-eukaryotic translation initiation factor 4E binding protein 1 (4EBP1); anti-phospho-4EBP1, Ser65; anti-eukaryotic translation initiation factor 4E (eiF4E); anti-muscle atrophy F box protein (Atrogin1); anti-muscle specific finger protein 1 (MuRF1); anti-microtubule-associated protein 1 light chain 3 beta (LC3B); anti-apoptosis-related cysteine peptidase 3 (caspase-3); anti-tubulin; anti-β-actin; and anti-glyceraldehyde-3-phosphate dehydrogenase (GAPDH), all from Proteintech (Wuhan, China). The membranes were then washed with Tris-buffered saline containing tween (TBST; Solarbio, Shanghai, China), and incubated with a 1:1000 dilution of a horseradish peroxidase (HRP)-conjugated goat anti-mouse IgG antibody (Beyotime, Shanghai, China) at 37 °C for 1 h. The proteins were visualized using Beyo ECL reagents (Beyotime, Shanghai, China) as the reference [22]. The intensity of the bands was quantified with a Pro Plus 6.0 Biological Image Analysis System.

### 2.7. Protein Synthesis Assay

To detect protein synthesis, we used a non-radioactive technical method called surface sensing of translation (SUnSET) [23]. In our study, 1 µg/mL of puromycin (Sigma, Aldrich, St. Louis, MO, USA) was added to each well, incubated for 40 min, and the total amount of puromycin incorporated into the peptide chain was assayed using anti-puromycin (Millipore, Billerica, MA, USA) and Western blotting methods to assess the rate of protein synthesis. The total protein concentration in the samples was determined using a BCA protein assay kit.

### 2.8. Data Analysis

The data were expressed as the mean ± SD and analyzed using one way ANOVA with SAS software. Multiple comparisons between the groups were performed with Tukey tests. Differences between treatments were considered statistically significant when *p* < 0.05.

## 3. Results

### 3.1. Isolation and Identification of SCs

**Figure 2:**
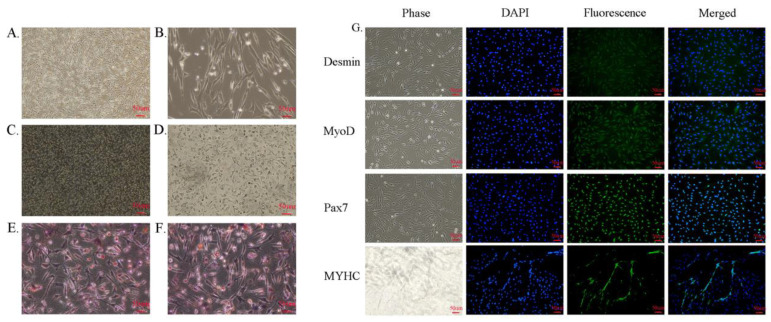
SCs identification results and test procedure. (**A**) SCs lipogenesis is induced for 1 d; (**B**) SCs morphology becomes flattened after lipogenesis induction. (**C**,**D**) SCs lipogenesis is induced for 14 d; (**E**,**F**) SCs lipogenesis is induced after oil red O staining, and intracellular lipid droplets are stained orange–red; (**G**) SCs immunofluorescence staining for anti-desmin, anti-MyoD, anti-Pax7, and anti-MYHC is indicated in green, and nuclei are stained blue with DAPI. Scale bar: 50 μm.

In the present study, we isolated SCs from 3 day old rabbit dorsal girdle muscles and identified SCs by the induced differentiation method and immunofluorescence before the start of the experiment. The results of induced differentiation show that SCs appear as distinct orange–red lipid droplets after oil red O staining, and SCs successfully differentiate into adipocytes. The results of immunofluorescence show that almost all anti-desmin, anti-MyoD, anti-Pax7, and anti-MYHC are co-localized with 4′,6-diamidino-2-phenylindole (DAPI)-labeled nucleic acids, which is evidence that these cells are SCs (Figure 2).

### 3.2. Effect of Lys on Proliferation, Apoptosis, and Autophagy in SCs (Experiment 1)

**Figure 3:**
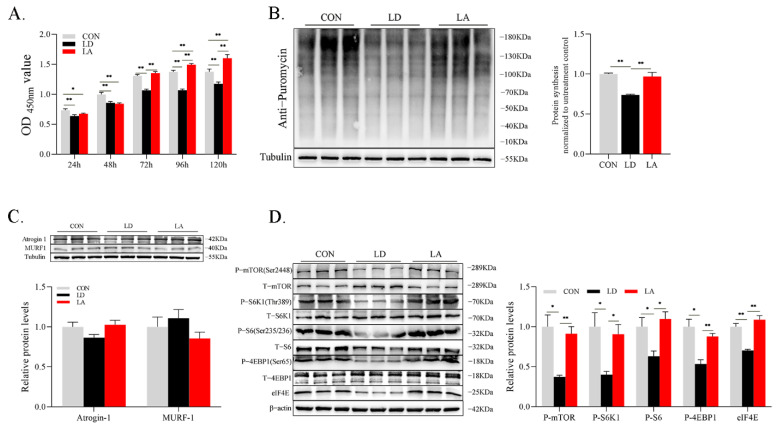
Lysine deficiency attenuates SCs proliferation, protein synthesis, and phosphorylation levels of the mTORC1 signaling pathway. (**A**) Cell proliferation; (**B**) representative images of cell protein synthesis levels with Western blotting; (**C**) representative images of protein ubiquitination level marker protein atrogin1 and MuRF1 expression levels with Western blotting; (**D**) representative images of phosphorylation levels of mTORC1 signaling pathway-related proteins with Western blotting. * *p* < 0.05, ** *p* < 0.01. The data shown represent mean ± SEM of three independent experiments (*n* = 3).

Compared with the control group, the SCs proliferation rate is significantly reduced between 24 and 120 h of continuous Lys deficiency, which is significantly restored when Lys is added (*p* < 0.05) (Figure 3A). The SUnSET detection technique was used to analyze the change of SCs protein synthesis rate. SCs protein synthesis is significantly inhibited after Lys deficiency for 120 h (*p* < 0.05), and significantly recovers with adequate Lys supplementation (*p* < 0.05) (Figure 3B). The expression of ubiquitin-related proteins (atrogin-1 and MuRF-1) is not affected by Lys deficiency and supplementation (*p* > 0.05) (Figure 3C). Lys content in the culture medium affects the protein expression related to the mTORC1 signaling pathway (Figure 3D). The levels of P-mTOR and its downstream proteins P-S6K1, P-S6, P-4EBP1, and eiF4E in the Lys deficiency group are significantly lower compared with the control. The addition of Lys in the Lys-deficit medium attenuates the Lys deficiency effect (*p* < 0.05).

**Figure 4:**
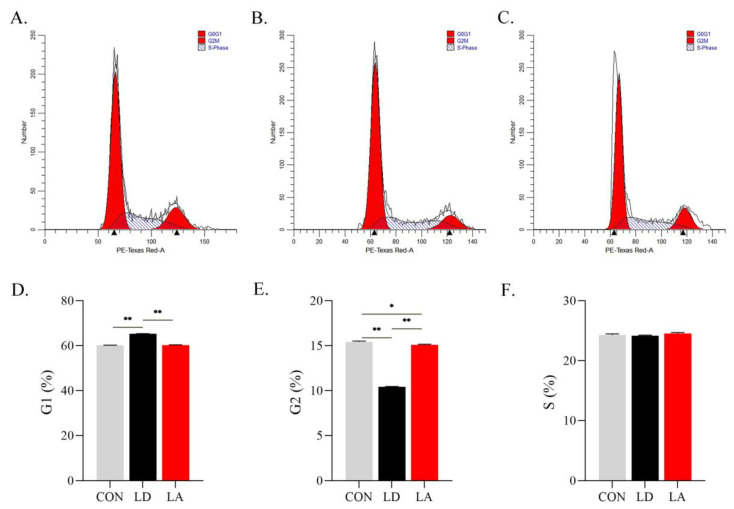
Lysine deficiency causes SCs cycle arrest. (**A**) CON; (**B**) LD; (**C**) LA; (**D**) G1 phase cell ratio (%); (**E**) G2 phase cell ratio (%); (**F**) S phase cell ratio (%). * *p* < 0.05, ** *p* < 0.01. The data shown represent mean ± SEM of three independent experiments (*n* = 3).

After Lys deficiency, the proportion of SCs in resting state/gap one (G0/G1) is significantly increased (*p* < 0.05), and in the second gap/mitotic phase (G2/M) is significantly decreased (*p* < 0.05) compared with the control group. Compared with the Lys deficiency group, the proportion of SCs in G0/G1 is significantly reduced (*p* < 0.05), and in G2/M is significantly increased (*p* < 0.05) in the Lys addition group. Lys level has no significant effect on cell proportion in the S phase (*p* > 0.05) (Figure 4A–F).

**Figure 5:**
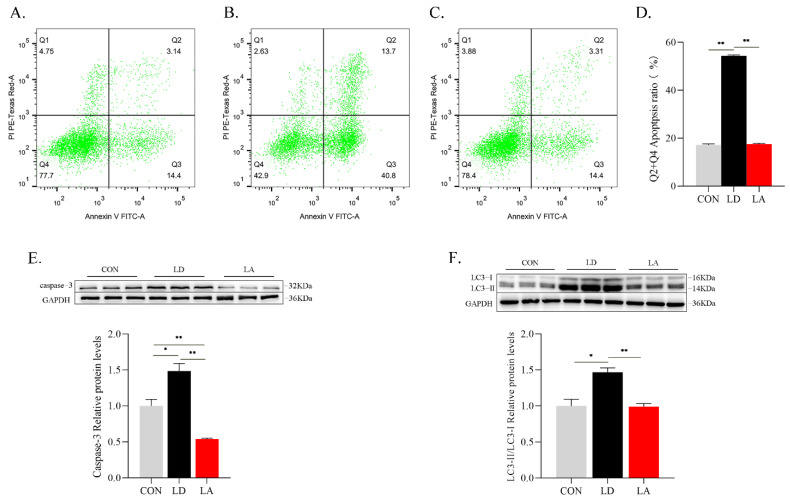
Lysine deficiency results in increased SCs apoptosis rate, apoptosis marker protein caspase-3 expression level and autophagy marker protein LC3-II/LC3-I ratio. (**A**) CON; (**B**) LD; (**C**) LA; (**D**) apoptosis rate (%); (**E**) representative images of caspase-3 protein expression levels with Western blotting; (**F**) representative images of LC3-II/LC3-I protein ratio with Western blotting. * *p* < 0.05, ** *p* < 0.01. The data shown represent mean ± SEM of three independent experiments (*n* = 3).

After 120 h of Lys deficiency, cell apoptosis rate and apoptotic protein caspase-3 expression in the Lys deficiency group are significantly increased compared with the control group (*p* < 0.05). Compared with the Lys deficiency group, cell apoptosis rate and caspase-3 protein expression decreases in the Lys addition group (*p* < 0.05) (Figure 5A–E). The LC3-II/LC3-I protein ratio is significantly increased compared with the control group (*p* < 0.05). The LC3-II/LC3-I protein ratio in the Lys addition group is significantly lower than in the Lys deficiency group (*p* < 0.05) (Figure 5F).

### 3.3. Function of the mTORC1 Signaling Pathway on Lys Regulating SCs Proliferation, Apoptosis, and Autophagy in SCs (Experiment 2)

**Figure 6:**
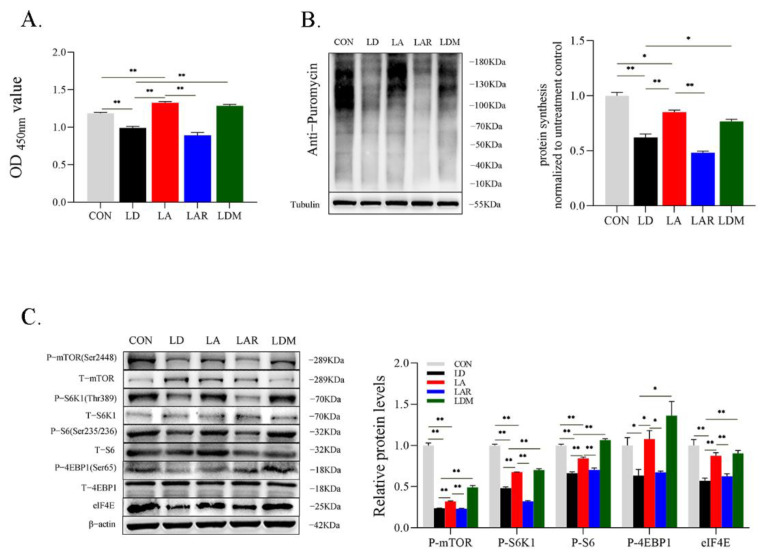
mTORC1 signaling pathway is involved in the regulation of SCs proliferation and protein synthesis by lysine. (**A**) Cell proliferation; (**B**) representative images of cell protein synthesis levels with Western blotting; (**C**) representative images of phosphorylation levels of mTORC1 signaling pathway-related proteins with Western blotting. * *p* < 0.05, ** *p* < 0.01. The data shown represent mean ± SEM of three independent experiments (*n* = 3).

Proliferation of SCs in the Lys addition + rapamycin group is significantly diminished compared to the Lys addition group (*p* < 0.05). MHY1485 treatment in absence of Lys significantly restores cell proliferation compared to the Lys deficiency group (*p* < 0.05) (Figure 6A). Rapamycin treatment in the Lys addition condition significantly decreases SCs protein synthesis compared to the Lys addition group (*p* < 0.05). In contrast, MHY1485 treatment in the Lys deficiency condition significantly increases SCs protein synthesis compared with the Lys deficiency group (*p* < 0.05) (Figure 6B). Rapamycin treatment in the Lys addition condition significantly decreases the protein expression of *p*-mTOR P-S6K1, P-S6, P-4EBP1, and eiF4E compared with the Lys addition group (*p* < 0.05). Compared with the Lys deficiency group, MHY1485 treatment in Lys deficiency significantly increases the protein expression of p-mTOR P-S6K1, P-S6, P-4EBP1, and eiF4E (*p* < 0.05) (Figure 6C).

**Figure 7:**
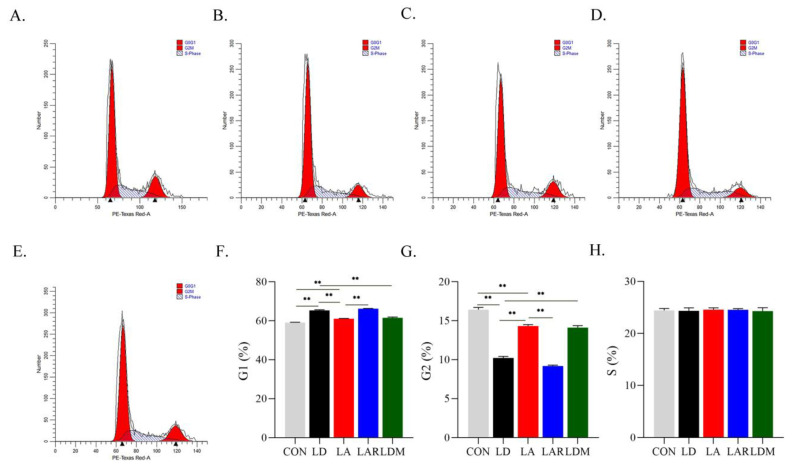
mTORC1 signaling pathway is involved in the regulation of SCs cycle by lysine. (**A**) CON; (**B**) LD; (**C**) LA; (**D**) LAR; (**E**) LDM; (**F**) G1 phase cell ratio (%); (**G**) G2 phase cell ratio (%); (**H**) S phase cell ratio (%), ** *p* < 0.01. The data shown represent mean ± SEM of three independent experiments (*n* = 3).

Rapamycin treatment in the Lys addition condition significantly increases the cell ratio in G0/G1, and decreases the cell ratio in G2/M compared with the Lys addition group (*p* < 0.05). In contrast, MHY1485 treatment in the Lys deficiency group significantly increases the cell ratio in G0/G1, and decreases the cell ratio in G2/M compared with the Lys deficiency group. The rapamycin and MHY1485 treatments have no significant effect on cell ratio in the S phase (*p* > 0.05) (Figure 7).

**Figure 8:**
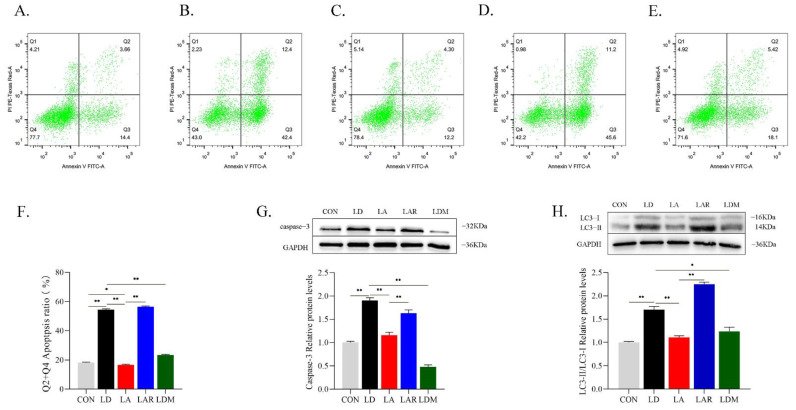
mTORC1 signaling pathway is involved in the regulation of apoptosis and autophagy by lysine. (**A**) CON; (**B**) LD; (**C**) LA; (**D**) LAR; (**E**) LDM; (**F**) apoptosis rate (%); (**G**) representative images of caspase-3 protein expression levels with Western blotting; (**H**) representative images of LC3-II/LC3-I protein ratio with Western blotting. * *p* < 0.05, ** *p* < 0.01. The data shown represent mean ± SEM of three independent experiments (*n* = 3).

Rapamycin treatment in the Lys addition condition significantly increases apoptosis rate and apoptotic protein caspase-3 expression compared with the Lys addition group (*p* < 0.05). In contrast, the MHY1485 treatment in the Lys deficiency condition significantly decreases the apoptosis rate and caspase-3 expression compared with the Lys deficiency group (*p* < 0.05) (Figure 8A–G). The Lys addition + rapamycin group experiences a significant increase in the LC3-II/LC3-I protein ratio compared with the Lys addition group (*p* < 0.05). In contrast, the Lys deficiency + MHY1485 group has a significant decrease in the LC3-II/LC3-I protein ratio compared with the Lys deficiency group (*p* < 0.05) (Figure 8H).

## 4. Discussion

In the current study, we investigated the effect of Lys deficiency or addition on SCs proliferation, apoptosis, and autophagy, and whether the mTORC1 signaling pathway is involved. Our data demonstrate that (1) Lys deficiency decreases SCs proliferation and protein synthesis, and induces cell autophagy and apoptosis, which is attenuated when Lys is added; (2) the activation of the mTORC1 signaling pathway attenuates the effect of Lys deficiency on SCs proliferation, protein synthesis, autophagy, and apoptosis; (3) the inhibition of the mTORC1 signaling pathway attenuates the effect of Lys addition on SCs proliferation, protein synthesis, autophagy, and apoptosis. The results suggest that Lys stimulates cell proliferation and inhibits cell apoptosis via the mTORC1 signaling pathway in SCs.

### 4.1. Lysine Promotes Muscle SC Proliferation

Lys plays an essential role in skeletal muscle growth by acting as a cell signaling molecule [24]. Similar to our previous study, the role of Lys in promoting skeletal muscle growth is demonstrated in experiments on animals [25]. However, despite the fact that both Lys and SCs are essential factors for skeletal muscle growth, the lack of association between them has been rarely reported. In the current study, we found that cell proliferation is diminished in Lys deprivation and is restored upon restoration of Lys levels, indicating that Lys acts as an agonist for cell mitosis [26,27]. This is also confirmed in the results of the cell cycle assays. The cell cycle of SCs is blocked and cell mitotic activity is diminished in the condition of Lys deprivation, and the effects are attenuated upon the restoration of Lys levels. Our results show that Lys deficiency fails to support cells to synthesize proteins in order to maintain cell proliferation and the cell cycle, causing reduction in cell viability and cell cycle arrest.

In addition, there is a large amount of protein synthesis during cell proliferation [28]. We examined the protein synthesis of SCs using the SUnSET method. The results show that protein synthesis of SCs is significantly inhibited in Lys deprivation, which is attenuated after Lys restoration. This result is in agreement with a previous study by Sato et al. [11]. Cellular protein accumulation is a dynamic process of synthesis and degradation [29]. Therefore, we also investigated the effect of Lys on the ubiquitination process of cellular protein degradation. The present results show that Lys deprivation has no significant effect on SCs expression of atrogin-1 and MuRF-1, which play an important role in the ubiquitination process. Our results imply that the cellular protein degradation process may not be affected by Lys content in a culture medium. However, Sato et al. found that the rates of myofibrillar protein degradation from the isolated rat muscles after fasting are markedly suppressed after administration of Lys [11]. These conflicting results suggest that the effect of Lys on cellular protein degradation may be related to cell starvation. Our study implies that Lys only increases cellular protein synthesis and has no effect on the degradation process.

### 4.2. Lysine Deprivation Stimulates Muscle SCs Autophagy and Apoptosis

Apoptosis and autophagy are two forms of programmed cell death [30], but autophagy and apoptosis are two distinct processes. In general, the occurrence of autophagy activates the ability of cells to overcome stress and maintain homeostasis in vivo [31]. Autophagy can counteract apoptosis by creating an environment conducive to cell survival. Light chain 3 is used as a marker of autophagy because it was identified as the first mammalian protein localized in the autophagosome membrane [32]. LC3-II is increased by conversion from type I and the ratio of LC3-II to LC3-I is correlated with the extent of autophagosome formation [33]. In the current study, we found a significant increase in LC3-II/LC3-I protein levels of SCs after Lys deprivation, which is attenuated after the restoration of Lys content. The results suggest that the disruption of cellular AA metabolism after Lys deprivation leads to enhanced cellular autophagic activity. Intracellular AA levels are maintained by autophagy. In nutrient deprivation conditions, cells strongly induce autophagy to compensate for cellular demands and restore the AA pool [34].

In line with a previous study [27], Lys deficiency causes SCs apoptosis. Apoptosis relative protein (caspase-3) is markedly increased in response to Lys starving [35], which may mediate cell apoptosis in SCs. These results are in line with the function of high cholesterol, indicating that high cholesterol induction can elevate the protein expression of caspase-3 and LC3-II, leading to the simultaneous occurrence of apoptosis and autophagy [36]. Controversy remains over the effects between autophagy and apoptosis in previous studies. Both inhibitory and stimulatory effects of autophagy on apoptosis are reported in studies of human non-small cell lung cancer, as well as substantial mutual interference between the proteins that regulate autophagy and apoptosis [37]. In our study, the contrary tendency of cell autophagy on apoptosis after Lys deprivation implies apoptosis initiation in SCs when Lys deprivation is caused by cell autophagy. Another possibility is that there are two processes involved, and not a single cause, which needs further validation.

### 4.3. Function of mTORC1 Signaling Pathway in Lys Regulating Cell Autophagy, Autophagy, and Apoptosis in SCs

mTORC1 is a conserved Ser/Thr kinase that is recognized as a major receptor for AA [38]. mTORC1 integrates a variety of extracellular signals such as nutrients, energy, and growth factors; participates in biological processes such as gene transcription, protein translation, and ribosome synthesis; and plays an extremely important role in cell growth and apoptosis [17]. In our previous study, a stimulative effect of Lys on mTORC1 signaling pathways in rabbit muscle was found [15], which is in line with the finding in SCs. Our results show that Lys deprivation inhibiting the mTORC1 signaling pathway decreases the expression of P-mTOR protein and other downstream proteins (P-S6K1, P-S6, P-4EBP1, and eiF4E), which is attenuated with Lys addition. The results suggest that the mTORC1 signaling pathway may be associated with the regulating of Lys in cell proliferation, autophagy, and apoptosis.

To further determine the potential role of the mTORC1 signaling pathway in SCs during Lys-regulated autophagy and apoptosis, the mTORC1 signaling pathway was blocked by rapamycin treatment in the condition of Lys addition, and the mTORC1 signaling pathway was activated by MHY1458 treatment in the condition of Lys deprivation. Activation or inhibition of the mTORC1 signaling pathway attenuates the effect of Lys deprivation and Lys addition, respectively, on cell proliferation, protein synthesis, and the cell cycle. Similar to previous studies, activation or inhibition of the mTORC1 signaling pathway regulates the rate of protein synthesis, which further affects cell proliferation [39,40]. These results demonstrate that the mTORC1 signaling pathway is essential for Lys mediating the SCs proliferation involved in numerous processes.

mTORC1 is an important suppressor of autophagy and a regulator of cell metabolism. The role of the mTORC1 signaling pathway and its involvement in regulation of apoptosis and autophagy remains controversial [41]. In one study, mTORC1 inhibition reduces cardiomyocyte apoptosis and promotes cardiomyocyte autophagy by modulating cross-talk between mTORC1 and endoplasmic reticulum stress pathways in chronic heart failure [42]. In the present experiment, activating the mTORC1 signaling pathway attenuates the stimulative effect of Lys deprivation on apoptosis and autophagy in SCs. Meanwhile, inhibition of the mTORC1 signaling pathway increases apoptosis and autophagy in SCs in the Lys addition. These results demonstrate that the mTORC1 signaling pathway is involved in the process of Lys regulating apoptosis and autophagy in SCs. In the development of neuromuscular diseases, the occurrence of excess apoptosis increases the loss in muscle fibers, and in the absence of effective primary treatment, interventions for muscle fiber apoptosis hold promise for yielding promising therapeutic strategies [43]. The development of human aging and muscle disease is often accompanied by loss in muscle mass. Autophagy is a degradation pathway of macromolecules and organelles during aging and muscle loss, and maintenance of skeletal muscle mass requires proper induction or accurate regulation of the autophagic process [44]. In association with the present study, Lys is able to act as a nutrient for muscle growth, and may play a role in slowing the development of muscle disease.

## 5. Conclusions

**Figure 9:**
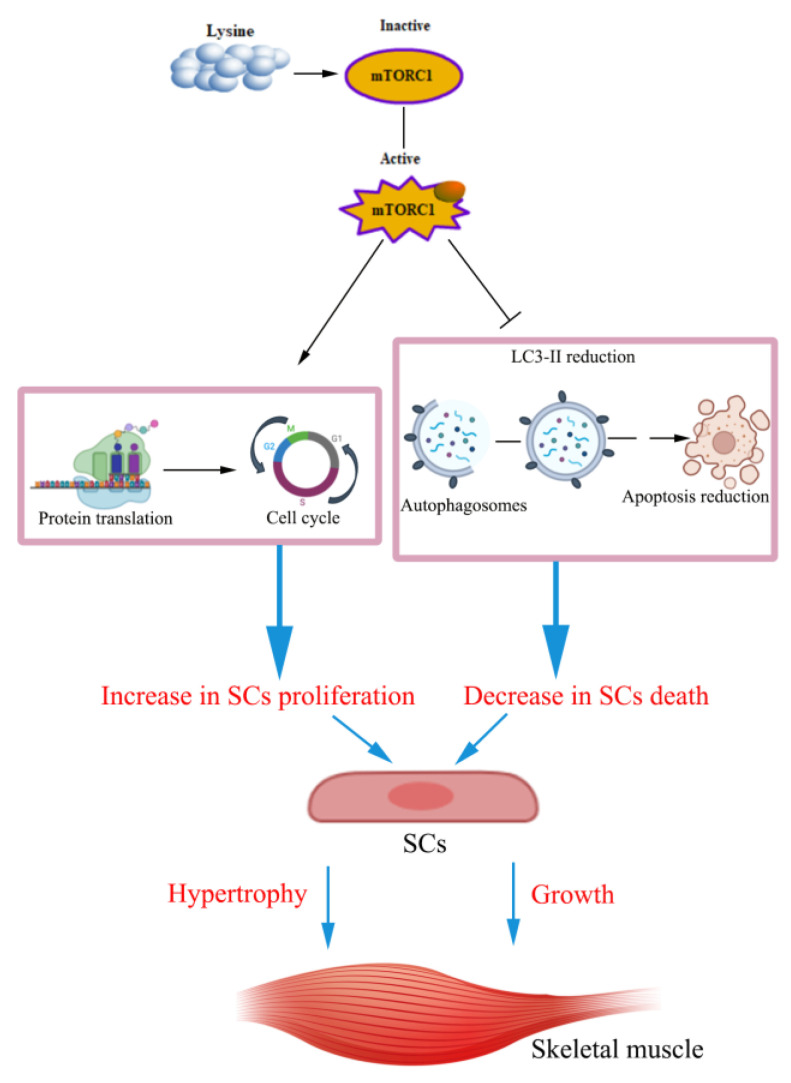
Proposed model of Lys on proliferation, apoptosis, and autophagy in SCs.

Our findings demonstrate that Lys improves the proliferation, cellular protein synthesis, and the cell cycle in SCs, and inhibits autophagy and apoptosis. Moreover, the mTORC1 signaling pathway mediates these processes. These effects are ultimately be reflected in the growth and hypertrophy of skeletal muscle (Figure 9).

## Data Availability

The data presented in this study are available on request from the corresponding author. The data are not publicly available due to follow-up studies are still in progress.
